# Lecturers' teaching competencies towards improving teaching and learning process in universities in Tanzania: Students’ perspectives

**DOI:** 10.1016/j.heliyon.2025.e41683

**Published:** 2025-01-03

**Authors:** Asia Mbwebwe Rubeba

**Affiliations:** The University of Dodoma-Tanzania, College of Education, Department of Educational Psychology and Curriculum Studies, P.O. Box 523, Dodoma, Tanzania

**Keywords:** Teaching competency, Pedagogical competency, Assessment skills, Content competency, Technological competency

## Abstract

This study examined lecturers' teaching competencies at universities in the attempt to improve the teaching and learning process by considering students' perspectives. Specifically, the study examined indicators of lecturers' teaching competencies in universities as well as establishing whether there is a relationship between students' perceived lecturers' teaching competencies and students' demographic parameters. The study used a cross-sectional survey design to generate data from 422 undergraduate students from three public universities in Tanzania using a questionnaire. Means, standard deviations, percentages and the independent T-test were used to analyse data. Experts and peer reviews were carried out to determine the validity of the study. The study's reliability was assessed using Cronbach Alpha in which content competency = 0.889, pedagogical competency = 0.809, assessment and evaluation skills = 0.701, and technological competency = 0.777 show acceptance of theoretical constructs. From the findings, students perceived content, pedagogical, assessment and technological competencies are key indicators of lecturers' teaching competencies that improve the teaching and learning process. Based on demographic factors, there was no relationship among the four variables. These findings imply that university lecturers are required to possess and master the perceived competencies and see how best they connect these competencies to students' needs, beliefs and aspirations during their teaching processes. Thus, in designing their lesson, lecturers have to consider the four teaching competencies, which in turn may improve students' performance. The study recommends a policy guideline by the Tanzania Commission for Universities for academicians to undergo training on assessment, pedagogy and technology. This will help lecturers to develop a common understanding of the teaching competencies and maintain teaching quality. Furthermore, universities should mobilize their resources and set aside enough funds to buy ICT facilities so that lecturers can easily integrate technology into their teaching processes so as to meet the current demand for technological advancement.

## 1 Introduction

Despite the multiple roles that are played by the lecturers at universities, the need for effective lecturers in teaching is inevitable. University is a place where think tanks are developed and founded, and therefore, there is no way teaching can be separated from other universities' core businesses like research, publications and community services. In that case, lecturers are expected to promote sufficient knowledge, skills, attitudes and values that students in the universities need to acquire and master to be used for different careers for different purposes. Early research on teaching competency has tried to identify the characteristics of effective lecturers by asking the students taught by them to list the characteristics of lecturers they considered to be good lecturers. The top six of twenty-five characteristics identified in this way by the Common-Wealth Teacher Training Study are adaptability, considerateness, enthusiasm, good judgment, honesty, and magnetism [1]. Hart [2] studied the high school students to list the characteristics which they liked the best. The liked most characteristics found among teachers frequently were teaching skills, being cheerful and good-natured, patience, not irritable, friendly, compassionate, not aloof, having interest in students, understanding them, being impartial, having no teacher's pets, and being fair in grading and marking. Subsequently, the same students were asked whether from whom they learnt most was the same as the one they liked the best, and if not, how the most effective teacher differed from the best effective teacher identified from the best liked. Four characteristics of the effective teacher identified from this list were: (1) meets greater demand of students, (2) has more teaching skills, (3) has more knowledge of subject matter, and (4) has better discipline. In this list, one competency is 'more teaching skill’ acquired through training.

In the 21st Century, studies on teaching competency indicate that lecturer competency in the teaching process is a multidimensional concept that measures various interrelated aspects, which include communication skills, subject matter expertise, lecturer attendance, teaching skills and lecturer attitude [3,4,5]. Muzenda [6] in his study indicates that there is a significant influence of lecturer subject knowledge, teaching skills, lecturer attendance and lecturer attitude on students' academic achievement. Muzenda [6] used 115 students as a sample size to determine lecturers' competency in students' academic achievement. From the findings it recommends the provision of consistent training to the lecturers to improve their teaching competency that may improve students’ academic performance.

Alqiawi & Ezzeldin [7] suggested a model for developing and assessing the competency of prospective teachers in faculties of education in Saudi Arabia. From their findings, the study revealed that academic competency (broad knowledge base and understanding of the taught subject), professional competency (ability to apply various strategies in teaching, using theories of teaching in an effective way, and ability to implement teaching methods in actual classroom situation) and personal competencies (values, attitudes, general intelligence, good morals, linguistic ability, ability to innovate and personal effectiveness of the person) are the key teachers’ competencies that should be inculcated in teacher education.

Various studies, including Akram et al. [8,9], Velasco [10], and William, Abdon and Mbepera [11], show different views on lecturers’ competencies in higher education. Akram et al. [9] for example, studied the technology integration in higher education during COVID-19 in Karachi-Pakistan. Specifically, the study was based on an assessment of online teaching competencies through the technological pedagogical content knowledge (TPACK) model. The findings revealed that teachers possessed adequate levels of knowledge across all the domains of TPACK. However, in determining the levels, content knowledge scored higher competency compared to technological knowledge which scored lower competency. The study suggests that relevant educational authorities and policymakers should enhance the technological competencies of teachers for quality online education. William, Abdon and Mbepera [11] in their study on e-Learning during the COVID-19 pandemic in Tanzanian Universities: Policy challenges and implications. It was observed from their study that lecturers and students were found to have limited knowledge of ICT usage. It was suggested from the study that the enhancement of the teaching and learning process through learning is possible if universities could offer capacity building to the academic staff and students.

Noteworthy, Kitula, Kireti, & Wambiya [12] in their study on the perceived efficacy of university lecturers in conducting assessments among selected universities in Tanzania observed that the literacy level of lecturers in educational assessment was found to be minimal as most lecturers were found not to be aware on some methods of assessment including peer and self-assessment despite their significance in the learning process. According to this study, it was observed that lecturers prefer mostly written tests which are simple to prepare and administer. However, their preferences could be due to limited knowledge of other aspects of assessment methods. Literature show that the lack of proper techniques in assessment may result in failure to enhance thinking ability among students [12,13] and this may lead to poor learning outcomes that has been identified by Mnubi [14] and Kira [15] as being one of the challenges facing higher education in Tanzania.

A common understanding and expectations of university students is that a university will enhance their academic and career prospects, but also provide opportunities to become self-independent and enjoy themselves [16,5]. It is the matter of fact that student-lecturer interaction, smooth communication, mutual support and understanding, play significant roles towards students' attainment [17] Despite that, there are discrepancies between what students’ expect to get from their lecturers based on their roles to play and the actual university life. For example, Lowe and Cook [16] reported that 41 % of their group expected lecturers to be more sympathetic and reassuring, and 35 % thought that lecturers would be more helpful and friendly.

Moreover, students perceive effective teaching as bringing their success in universities and places learning outcomes as paramount [18]. These learning outcomes include; knowledge, skills, depth of lecture, teachers' feedback of their work, class notes and reading materials. Studies indicate that without instructors' ability to connect learning outcomes visa-viz their expectations, aspirations and beliefs; there is a likelihood of students to be stressed, perform poorly in academics, and drop out from studies (Yorke,. and Longden, 2004: Dolence & Norris 1995 cited in Malechwanzi., Lei.,& Wang [19] and in the future vicious circle may be created as some of them are expected to be lecturers if succeed to complete their studies. Instructors act as role models and students learn from them. Henceforth, instructors need to be updated on various competencies and be able to align with students' needs and which will make students more engaged in due process of teaching and learning. Previous studies on lecturers' competency were based on the integration of technology in teaching and learning process [8,9,11], other studies were focused on lecturers' competency in the assessment and evaluation of students' activities [12,13]. Most of these studies have been focusing on academic staff views, with minimal attention on students' views who are the key beneficiaries of the teaching and learning process and thus any changes may influence their knowledge base either positively or negatively. It is from this angle, therefore, the study examined ′lecturers' teaching competencies on improving teaching and learning process in universities in Tanzania by focusing on students’ views.

### 1.1 Westera’ Model of Teacher competency

**Fig. 1:**
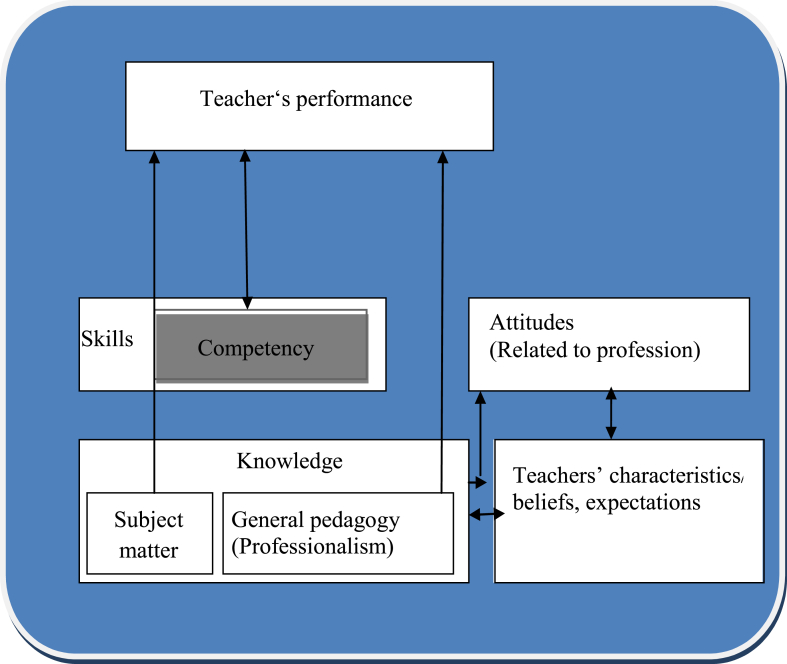
Adopted Westera's model of Teacher's competency [20].

This model was developed by Westera in 2001. The model explains that teacher competency directly links teachers’ knowledge; that is, subject matter and general pedagogy, characteristics and attitudes. Hence, this model assumes that teacher competency comprises the subject matter and general pedagogy (see Fig. 1).

Under this model, the subject matter is linked to the general pedagogy, which includes knowledge about classroom organisation and management, general knowledge of lesson structure, and general methods of teaching. These ideas corroborate with Shulman [21], Mishra and Koehler [22] who discussed various kinds of knowledge. According to Shulman [21] there are three kinds of knowledge: content knowledge, pedagogical content knowledge (PCK), and curriculum knowledge. Content knowledge refers to the amount and organization of knowledge in the mind of the teacher. Mishra and Koehler [22] modified the Shulman model by adding the component of technological knowledge and emphasized that teachers are required to be aware of the connections and ways to produce effective teaching for students to understand the concepts taught and apply the knowledge in the real life. Westera [20] stresses that teachers must not only be capable of defining the content or concepts for learners but they must also be able to explain why and how these concepts relate to other concepts or content, as well as explain why a particular proposition is deemed warranted. The knowledge of pedagogical content goes beyond the knowledge of the subject matter to the dimension of knowledge of the subject matter for teaching. Finally, curricular knowledge is the knowledge of the full range of programmes designed for the teaching of particular subjects and topics at a given level. Westera [20] maintains that the use of particular curriculum or programme materials in particular circumstances requires instructors to have a variety of teaching materials related to those programmes and a set of characteristics that serve both as indications and contra-indications.

## 2 Research objectives

The study employed the following objectives in order to generate findings.

i. Examine the indicators of lecturers' teaching competencies in universities that could improve the teaching and learning process.
ii. Establish the relationship between students' perceived lecturers' teaching competencies in universities and their demographic parameters.

## 3 Methodology

This study was conducted in Tanzanian public universities. The study was conducted in Tanzanian universities because few studies on lecturers' teaching competencies were observed during the review of the literature and in those studies that were reviewed most of them are focusing on secondary and primary school teachers' competency with little interest at universities. Therefore, conducting a study on assessing the lecturers' teaching competencies in Universities in Tanzania using students’ perspectives was timely.

Notably, the study was conducted in Dar es Salaam, Morogoro and Dodoma Regions with a major focus on big universities. Purposive sampling was used to select these universities under study. The researcher believed that large number of students with different characteristics might be found in the selected universities. In order to obtain the sample size of the study, stratified proportional and simple random sampling techniques were used to obtain 422 third and fourth year undergraduate students from different programmes. According to Altunışık et al. (2004) as cited by Akram et al. [9], show that the size of the sample is required to be between 30 and 500 at a 5 % confidence level with 95 % significance level. Again, Tabachnick & Fidell, (1996) as cited by VanVoorhis & Morgan [23] stated that, if the study uses factor analysis to determine the factors loading, the rule of thumb has to be used in order to get the sample size and the sample size has to be around 300 or 500 cases in order to have good representatives of the respondents in that study. Therefore, the sample size of 422 is found to be adequate for conducting this study. The population of the study involves all students from various colleges and schools including Education, Natural and Applied Science, Humanities and Social Sciences, Business Studies and Agriculture. The study assumed that these students have stayed longer in the universities and interact more with lecturers as compared to other colleagues (first and second year undergraduate students). Henceforth, they might be well informed about the way their lecturers perform their teaching activities in relation to their competency levels of instructions. Furthermore, another reason for choosing third and fourth year was that some of the courses are extensions of the previous courses, which create higher student-lecturer interaction. Additionally, some lecturers are teaching more than one course in the same class, which results in higher contact between students and lecturer. Therefore, the study expected that their longer stay at universities would create more understanding of their lecturers as compared to first and second-year students studying at the same universities; as a result of this, their perceptions would be different from their counterparts. Besides, in most cases year 1 is for familiarization or orientation of university life while in year two of their academic studies is when now the student starts to settle for academic purposes (This is from my experience as a university lecturer with fourteen (14) years of teaching at various universities in my country). Thus, the selection created a greater opportunity for the study to attain more respondents and information and hence promoted an opportunity for enriching both the validity and reliability of its findings. Furthermore, cross-sectional survey design was used as the design allows the data to be collected at the same time from many different individuals and levels. Descriptive and inferential statistics were used to analyse quantitative data. Principle Component Analysis followed by Exploratory Factor Analysis was used to find out the factor loadings which used as indicators of lecturers teaching competencies as perceived by students. In order to find out the content and construct validity of the study, questionnaires were sent to two experts of educational research and measurement for validation purposes. The questionnaires were attached with the problem of the study, the specific objectives and the rationale for undertaking the study. From the questionnaire, it was observed that some items had language ambiguities which were later modified by the experts and other items were repeated. These repeated items were removed from the study. Prior to the actual data collection, 51 items were prepared to be used for measuring students' perceived lecturers teaching competencies, but after the review process by experts and the performed commonalities after extraction, 12 items were removed from the study and remained with 39 items for study purposes. Kaiser-Meyer-Olkin (KMO) measure of sampling adequacy and Bartlett's test were performed to comply with Factor Analysis, (See Table 1).

**Table 1:** Kaiser-Meyer-Olkin measure of sampling adequacy and Bartlett's test.

**Kaiser-Meyer-Olkin Measure of Sampling Adequacy**		0.812
**Bartlett's Test of Sphericity**	Chi-square value	1245.575
	Df	190
	P-Value	<0.001

Table 1 presents an analysis revealing the obtained KMO value was 0.812, indicating a high sampling adequacy for factor analysis which is beyond the cut-off point of 0.5. Besides, the recorded significant value of Bartlett's tests at <0.001 implies that the original R-matrix is significantly different from an identity matrix. These findings suggest some correlations between test variables of students' perceived lecturers' teaching competencies and the data are suitable for factor analysis, thus they could be used in determining the content and construct validity of the instrument. Noteworthy, Cronbach Alpha was used to determine the reliability of the study in which content competency = 0.899, pedagogical = 0.809, Assessment and evaluation skills = 0.701, and technological competency = 0.777, which shows acceptance of theoretical constructs. Therefore, these data indicates that the items in the instrument were reliable. Ethical considerations were strictly followed to safeguard the privacy and security of respondents, ensuring they could answer the questions freely. Ethical protection was done through seeking permission from the University of Dar-es-Salaam (Reference Number-UDOM/GR/209 VOL. 1/37), Sokoine University of Agriculture (Reference Number-UDOM/GR/209 VOL. 1/38), Mzumbe University (Reference Number- UDOM/GR/209 VOL. 1/39) and Dar-es- Salaam University College of Education (Reference Number- UDOM/GR/209 VOL. 1/36). Permission to collect data was granted by the respective universities as follows; University of Dar-es- Salaam (Reference Number-AB3/31), Sokoine University of Agriculture (Reference Number-SUA/ADM/R.1/8VOL.IV/214), Mzumbe University (Reference Number-MU/R.2/1/VOL.II/174) and Dar-es-Salaam University College of Education (Reference Number-KE 354/401/01 “A”). The researcher was introduced to the college principals, deans, heads of departments and students respectively. Informed consent was obtained from all participants for the study. Furthermore, bracketing was done in order to provide fairness and justice during data collection, writing and reporting of the information.

## 4 Research findings and discussion

The findings and discussions of the findings were based on the stated objectives, as detailed below.

### 4.1 Indicators of lecturers teaching competencies that improve teaching and learning process in universities

Indicators of lecturers’ teaching competencies are categorised into four dimensions: Content, pedagogical competency, technological competency and assessment and evaluation skills.

#### 4.1.1 Lecturers’ content competency

Content competencies comprised seven (7) indicators as observed in Table 2 below (n = 422, mean cut-off point = 4.00). This means that an indicator which scored the mean above the cut-off point is described as high, and the indicator which scored the mean below the cut-off point is described as low competencies.

**Table 2:** Indicators for measuring Lecturers’ Content Competency.

Statement	Mean $\left(\bar{x}\right)$	Standard deviation	Not at all	Very little	A Little	Much	Very much
			f (%)	f (%)	f (%)	f (%)	f (%)
My instructors possess up-to-date knowledge and skills in his/her subject matter.	4.15	0.941	8(1.9)	19(4.5)	55(13.0)	161(38.2)	179 (42.4)
My instructors define prerequisite knowledge of the course from the outset.	4.04	0.936	4(0.9)	27(6.4)	72(17.1)	166(39.3)	153(36.3)
My instructors join students' previous learning experience with present knowledge.	3.99	1.078	18(4.3)	24(5.7)	66(15.6)	149(35.3)	165(39.1)
My instructors have ability to relate the course content to students' field of study.	4.14	1.038	15(3.6)	21(5.0)	49(11.6)	144(34.1)	193(45.7)
My instructors interpret the course contents from theoretical grounds to real life.	4.05	1.063	12(2.8)	29(6.9)	70(16.6)	128(30.3)	183(43.4)
My instructors transfer knowledge to real life.	4.08	1.045	12(2.8)	28(6.6)	58(13.7)	143(33.9)	180(42.7)
My instructors motivate students to be creative and innovative	3.97	1.153	21(5.0)	33(7.8)	62(14.7)	129(30.6)	177(41.9)

From Table 2 the findings show that lecturers possess of up to date subject matter knowledge and skills (mean = 4.15), instructors' ability to relate the course content to students' field of study had a mean of 4.14, ability of the instructor to transfer knowledge to real-life had a mean of 4.08, ability of the instructor to interpret the course contents from theoretical grounds to real life had a mean of 4.05, instructors join students' previous learning experience with present knowledge (mean = 3.99) and instructors motivate students to be creative and innovative (mean = 3.97). Among the 7 indicators of content competency, 5 indicators scored the mean above the cut-off point, and 2 scored below the mean. These can be summarized as the observed higher learning institutions had instructors with higher content competency due to most (71.43 %) of indicators that measure content competency to score the mean, which is above the cut-off point. The findings of this study are in agreement with the previous study by Akram et al. [9] who observed that content knowledge competency scored higher compared to other competencies in the Technological, Pedagogical, Content Knowledge (TPACK) model. These findings concur with scholars’ view [[4], [24], [25]] that apart from other competencies that an instructor should possess, content competency is a key towards improving his/her teaching. Instructors are expected to promote sufficient knowledge, skills, attitudes and values that students in the universities need to acquire and master to be used for different careers for different purposes.

#### 4.1.2 Lecturers’ pedagogical competency

Pedagogical competency comprised eight (8) indicators as observed in Table 3 below (n = 422, mean cut off point = 4.00). This means that the indicator which scored the mean above the cut-off point is described as high and the indicator which scored the mean below the cut-off point is described as low competency.

**Table 3:** Indicators for measuring lecturers’ pedagogical competency.

**Statements**	**Mean**	**Standard Deviation**	**Not at all f (%)**	**Very little f (%)**	**A little f (%)**	**Much f (%)**	**Very Much f (%)**
My instructors vary teaching methods to account for individual differences.	3.77	1.093	18 (4.2 %)	45 (10.7 %)	73 (17.3 %)	171(40.5 %)	115 (27.3 %)
My instructors determine teaching methods suitable for one's course	3.92	1.115	12(2.8 %)	18(4.3 %)	64(15.2 %)	151(35.8 %)	177(41.9 %)
My instructors plan for the mode of presentation (lecture, seminar) according to students' characteristics	4.1	0.999	22(5.2 %)	30(7.1 %)	57(13.5 %)	162(38.4 %)	151(35.8 %)
My instructors chair seminars and presentations	3.75	1.289	17(4.0 %)	42(10.0 %)	68(16.1 %)	136(32.2 %)	159(37.7 %)
My instructors are ready to learn from their students	3.9	1.136	38(9.0 %)	41(9.7 %)	62(14.7 %0	127(30.1 %)	153(36.2 %)
My instructors use modern learning methods to motivate continuous learning	3.82	1.074	7(1.7 %)	51(12.1 %)	94(22.3 %)	131(31.0 %)	139 (32.8 %)
My instructors apply strategies of cooperative learning to encourage teamwork	3.96	1.822	19(4.5 %)	28(6.6 %)	74(17.5 %)	159(37.7 %)	142(33.6 %)
My instructors organize the learning environment in the classroom in a way that reassures tranquility and induces feelings of security among students	3.8	1.142	24(5.7 %)	32(7.6 %)	85(20.1 %)	144(34.1 %)	137(32.5 %)

Table 3 shows the indicators for measuring pedagogical competency with a sample size of 422. The findings indicate that the ability of instructors to plan the mode of presentation (lecture, seminar) according to students' characteristics scored higher marks compared to other indicators, such as instructors' ability to apply strategies of cooperative learning to encourage teamwork scored the mean of 3.96, ability of instructors to determine teaching method suitable for one's course scored mean of 3.92, ability of the instructors to be ready to learn from their students scored 3.9, ability of instructors to use modern learning methods to motivate continuous learning scored the mean of 3.82, ability of the instructors to organize the learning environment in the classroom in a way that reassures tranquility and induces feelings of security among students scored mean of 3.8, ability of instructors to vary teaching methods to account for individual differences scored mean of 3.77. In contrast, the ability of instructors to chair seminars and presentations scored the mean of 3.75. These findings underscore the lecturer's planned mode of presentation but failed to chair seminars and presentations. This might be due to the number of students in the class and other related factors, such as universities having limited seminar rooms and few lecturers who can serve a large number of students in one class.

Literatures support the notion that pedagogical competency is a key criterion for quality education [29, 9]. Under this, lecturers are obliged to understand how and when to employ various teaching pedagogies based on nature of the subject they instruct, students' characteristics and the nature of the class [20]. Along with the varying degrees (see Table 2) in employing such pedagogy [26], studies indicate that lecturers should develop pedagogical knowledge and skills for promoting students’ knowledge acquisitions [21,27]. Therefore, the study suggests universities provide frequent seminars and workshops to their lecturers to improve pedagogical skills.

#### 4.1.3 Lecturers’ assessment and evaluation skills

Assessment and evaluation skills comprised of five (5) indicators as observed in Table 4 below (n = 422, mean cut-off point = 4.00). This means that the indicator which scored the mean above the cut-off point is described as high, and the indicator which scored the mean below the cut-off point is described as low competencies.

**Table 4:** Indicators for measuring lecturers ‘assessment and evaluation skills.

**Statements**	**Mean**	**Standard Deviation**	**Not at all f(%)**	**Very little f(%)**	**A little f(%)**	**Much f(%)**	**Very Much f(%)**
My instructors use varied evaluation methods suitable to behavioural objectives of the course	3.75	1.044	16(3.8 %)	34(8.1 %)	96(22.7 %)	171(40.3 %)	105(24.9 %)
My instructors are very fair in grading of assignments and tests	3.86	1.084	18(4.3 %)	30(7.1 %)	83(19.7 %)	153(36.3 %)	138(32.7 %)
My instructors evaluate students through meaningful and authentic situations	3.91	1.037	14(3.3 %)	30(7.1 %)	73(17.3 %)	171(40.5 %)	135(31.8 %)
My instructors have capacity to provide timely feedback on assignments and tests	3.87	1.101	15(5.2 %)	42(10.0 %)	73(17.3 %)	149(35.3 %)	143(33.9 %)
My instructors assign suitable and enough time for evaluation	3.89	1.092	24(5.7 %)	25(5.9 %)	88(20.9 %)	163(38.6 %)	122(28.9 %)

The findings in Table 4 show that all indicators for measuring assessment and evaluation skills scored below the cut-off points. For example, the ability of instructors to evaluate students, through meaningful and authentic situations, scored the mean of 3.91; ability of instructors to assign suitable and enough time for evaluation, scored the mean of 3.89; the ability of instructors to have the capacity to provide timely feedback on assignments and tests, scored the mean of 3.87, the ability of instructors to be fair in the grading of assignments and tests, scored the mean of 3.86 while the ability of instructors to use varied evaluation methods suitable to behavioural objectives of the course scored the mean of 3.75. It is clearly observed from the findings that assessment and evaluation was perceived lower by the students. The reasons are due to poor test construction and administration, unfair marking and limited timely feedback given to the students which do not encourage them to lean more [12,28,13]. The lower knowledge and skills of lecturers in assessing and evaluating teaching and learning activities might be due to different orientations among lecturers. There are those who are professional teachers whose one of the courses during teacher education training was educational measurement, assessment and evaluation. Within this course, prospective teachers are exposed to various assessment and evaluation tools both traditional and authentic/performance-based assessment. Another group of the lecturers are those with specialised subjects/subject experts and programmes. Assessment and evaluation course is not among of their courses during undergraduate and postgraduate programmes. As a result, they gain knowledge through seminars and workshops which are not guaranteed. However, studies indicates that even those lecturers who undergone teaching programmes in their due course are less effective in implementing the knowledge and skills obtained during their studies [29,12,13] in that case time to time professional development is vital.

#### 4.1.4 Lecturers’ technological skills

Technological skills comprised of three (3) indicators as observed in Table 5 below (n = 422, mean cut-off point = 4.00). This means that the indicator which scored the mean above the cut-off point is described as high and the indicator which scored the mean below the cut-off point is described as low competencies.

**Table 5:** Indicators for measuring lecturers’ technological competency.

Statements	**Mean**	**Standard Deviation**	**Not at all f(%)**	**Very little f(%)**	**A little f(%)**	**Much f(%)**	**Very Much f(%)**
My instructors use and employ ideas in scientific articles to improve one's course.	3.73	1.093	21(5.0 %)	39(9.2 %)	81(19.2 %)	173(41.0 %)	108(25.6 %)
My instructors Integrate information and communication technology in teaching and learning process.	3.92	1.083	18(4.3 %)	26(6.2 %)	79(18.7 %)	150(35.5 %)	149(35.3 %)
My instructors use technological and educational techniques to strengthen one's teaching skills and facilitate the process of learning	3.89	1.071	18(4.3 %)	31(7.3 %)	67(18.9 %)	171(40.5 %)	135(32.0 %)

Even though technological competency is one of the competencies that are needed to be acquired by university lecturers, the findings in Table 5 indicate that none-of the indicators that measures Lecturers' technological competency scored higher mean. For example, the ability of instructors to integrate information and communication technology in the teaching and learning process scored the mean of 3.92, the ability of instructors to use technological and educational techniques to strengthen one's teaching skills and facilitate the process of learning, scored the mean of 3.89 while, the ability of instructors to use and employ ideas in scientific articles to improve one's course, scored the mean of 3.73. These findings are indications that there is minimal integration of technologies during teaching and learning processes in universities. The reasons might be lack of technological resources among universities, both hardware and software, limited knowledge among lecturers which hinders the effective implementation of the technology. This is why Barineka [30] observed that instructors prefer the use of textbooks and reference books over digital technologies. Besides, Mishra and Koehler [22] noted that integrating technology into learning has added complexity to the fundamental knowledge of what constitutes the teachers' professional knowledge base. Nevertheless, writing on the importance of using technology, Mishra and Koehler [22] emphasize that technological knowledge needs to be included in addition to the content and pedagogical knowledge for the teaching and learning process. They added that it is high time that instructors are equipped with knowledge about the technological tools that could be used during teaching and learning. These technological tools include computer-based (Information & Communications Technology, Content-based Instructions, Computer-Assisted Instructions), Mobile-based (laptops, tablets, mobile phones), online learning and teaching tools (flipped classroom) and multimedia technologies (videos). However, under this digitization era, lecturers might face two key challenges as revealed by Channa and Sahito, [31]. These include development of their digital skills, and the creation of teaching activities that provide all students with the necessary skills (page. 2930). Besides, Akram et al. [8] in their study observed that the slow speed of the internet, load shedding, lack of infrastructure, online teaching experience, and training were reported as the main obstacles that hinder teachers from effective integration of Information Communication and Technology (ICT) into their teaching practices. Similar observations were made by William, Abdon and Mbepera [11] in their study on e-learning during the COVID-19 pandemic in Tanzanian Universities: Policy challenges and implications. Their study revealed that limited knowledge of the use of e-learning facilities by academic staff and students was a barrier to the integration of ICT in the teaching and learning processes. Unless these challenges are resolved, lecturers cannot compete in the digital world whereby lecturers employ modern digital pedagogical skills in teaching and learning process.

### 4.2 The relationship between students' demographic parameters and their perceptions on lecturers’ teaching competencies

Students' perceptions of lecturers’ teaching competency based on demographic parameters (Sex and year of study) were categorised in four dimensions: content, pedagogical competency, technological competency and assessment and evaluation skills). T-test for independent variables using 95 % confidence interval with 0.05 as a significance level was applied to compare the means among the demographic parameters. Results from students are explicitly shown in Table 6, Table 7, Table 8, Table 9 as follows.

**Table 6:** The relationship between perceptions of students on lecturers’ content competency based on sex and year of study.

	**Perception of the students**	**Indicators of Content Competency**			***t*-test Independent**		
		n	Mean	Standard deviation	T	Df	Sig
**Sex of the students**	Male	264	28.8826	4.90959	2.609	420	0.454
	Female	158	27.5886	4.96629	2.601	327.487	
**Year of study**	3rd year	254	28.5433	5.00001	0.738	420	0.732
	4th year	168	28.1786	4.91767	0.741	361.798

**Table 7:** Perceptions of students on lecturers’ teaching competencies based on sex and year of study when measured by indicators of pedagogical competency.

	Perceptions of the students	Indicators of pedagogical Competency			*t*-test Independent		
		N	Mean	Standard deviation	t	Df	Sig
Sex of the students	Male	264	11.4053	2.67479	−0.714	420	0.626
	Female	158	11.6013	2.81927	−0.704	316.831	
Year of study	3rd year	254	11.4252	2.72891	−0.495	420	0.881
	4th year	168	11.5595	2.73304	−0.495	357.308

**Table 8:** Perceptions of Students on Lecturers’ Teaching Competencies based on sex and year of study when measured by Indicators of Assessment and Evaluation Skills.

	Perceptions of the students	Indicators of Assessment and evaluation skills			*t*-test Independent		
		N	Mean	Standard deviation	T	Df	Sig
Sex of the students	Male	263	11.5133	4.17449	0.929	419	0.861
	Female	158	11.1646	2.83261	1.019	412.829	
Year of study	3rd year	253	11.3794	4.33497	−0.020	419	0.419
	4th year	168	11.3869	2.56864	−0.022	414.201

**Table 9:** The relationship between perceptions of students on lecturers’ teaching competencies based on sex and year of study when measured by indicators of technological competency.

	Perceptions of the students	Indicators of Lecturers Technological Competency			*t*-test Independent		
		N	Mean	Standard deviation	T	Df	Sig
Sex of the students	Male	264	77.4508	12.53487	0.612	420	0.875
	Female	158	76.6899	12.07508	0.618	340.312	
Year of study	3rd year	254	77.0866	12.69404	−0.162	420	0.949
	4th year	168	77.2857	11.86256	−0.164	374.110	

#### 4.2.1 Students' perceptions on lecturers’ teaching competency measured by indicators of content competency based on sex and year of study

Table 6 shows that the p value for both sex and year of study (p > 0.05) is greater than 0.05, then the Null hypothesis is not be rejected. Since the P value is greater than 0.05 confidence level, there is no statistically significant relationship between the two means (P = 0.454 i. e P > 0.05) for sex and (P = 0.732, i.e., P > 0.05) for the year of study. The findings show that there is no statistical relationship between male and female students' perceptions of lecturers' teaching competencies when measured by using content competency as well as the number of years of study. Furthermore, the mean scores of male students show higher, i.e., mean = 28.88, standard deviation = 4.91, t = 2.609) compared to female students (Mean = 27.59, standard deviation = 4.97, t = 2.601). Again, the mean scores for 3rd year students was slightly higher than the mean scores of 4th year students, i.e., (3rd year students' mean score = 28.54, Standard deviation = 5.00, t = 0 0.738 and 4th year students' mean scores = 28.18, standard deviation = 4.92, t = 0.741). This indicates that males' students do not share the same perceptions as females' students; likewise, 3rd year students do not share the same perceptions as 4th year students. Each group has a distinct perception of lecturers' teaching competencies, particularly in terms of content knowledge. Instructor's competency is a key determinant of students' performance [24, 20, 9]. Students' perceptions of lecturers' content competency, whether positive or negative, have a direct influence towards students' academic success. Knowledge about the subject is equally important since knowledge delivery is greatly influenced by the nature of students [32]. Female students require different treatment from their male counterparts [32]. This could be the reason why students' perceive differently about instructors teaching competency based on the content part. Besides, students would need instructors who handle them properly, regardless of their sex as well as their level of studies. Thus, instructors are required to be open-minded during instructions as some students may lose focus and, consequently, fail to comprehend the learned materials.

### 4.3 Perceptions of students on lecturers’ teaching competencies based on sex and year of study when measured by indicators of pedagogical competency

Table 7 shows that the p value for both sex and year of study (p > 0.05) is greater than 0.05, thus the Null hypothesis should not be rejected. Since the P value is greater than 0.05, the confidence level, hence it indicates that there is no statistically significant relationship exists between the two means (P = 0. 626 i. e P > 0.05) for sex and (P = 0. 881, i.e., P > 0.05) for the year of study. The findings show that there is no statistical relationship between male and female students' perceptions of lecturers' teaching competencies when measured by using pedagogical competency as well as the number of years of study. Furthermore, the mean scores of male students show lower, i.e., (Mean = 11.41, standard deviation = 2.67, t = −0.714) compared to female students (Mean = 11.60, standard deviation = 2.82, t = −0.704). Again, the mean scores for 3rd year students was slightly lower than the mean scores of 4th year students, i.e., (3rd year students' mean score = 11.43, Standard deviation = 2.73, t = −0.495 and 4th year students' mean scores = 11.56, standard deviation = 2.73, t = −0.495). The standard deviation is 2.73, with a t-value of −0.495 and a mean score of 11.56 for fourth-year students, also with a standard deviation of 2.73. This suggests that male and female students have differing perceptions, as do third-year and fourth-year students. Each individual group has different perception of lecturers teaching competencies regarding pedagogical competency. Arslantaş (2011) cited in Yilmaz and Tinmaz [33] observed that male students had higher perceptions than female students of lecturers’ competencies in using teaching strategies, methods and techniques.

### 4.4 Perceptions of students on lecturers’ teaching competencies based on sex and year of study when measured by indicators of assessment and evaluation skills

The results in Table 8 show that the p value for both sex and year of study (p > 0.05) is greater than 0.05 then the Null hypothesis should not be rejected. Since the P value is greater than 0.05 confidence level, it is conclude that there is no statistically significant relationship existing between the two means (P = 0.861, i.e., P > 0.05) for sex and (P = 0.419, i.e., P > 0.05) for the year of study. The findings it shows that there is no statistical relationship between male and female students' perceptions in lecturers' teaching competencies when measured by using the dimensions of assessment and evaluation skills. Similarly, the number of years of study shows no statistical significance relationship observed. Furthermore, the mean scores of male students show higher, i.e., (Mean = 11.5133, standard deviation = 4.17449, t = 0.929) compared to female students (Mean = 11.1646, standard deviation = 2.83261, t = 1.019). Again, the mean scores for 3rd year students show slightly lower than the mean scores of 4th year students, i.e., (3rd year students' mean score = 11.3794, Standard deviation = 4.33497, t = −0.020 and 4th year students' mean scores = 11.3869, standard deviation = 2.56864, t = −0.022). This indicates that male students do not share the same perceptions as female students; likewise, 3rd year students do not share the same perceptions as 4th year students. Each group has different perceptions of lecturers' teaching competencies as measured by the dimension of assessment and evaluation skills. The current study agrees with other studies [32,33], who studied students' perception of lecturers' measurement and evaluation competencies. The findings from the studies revealed that there was a significant difference in views depending on gender. However, the only difference between the current study and these previous studies was based on the type of gender whereby the current study observed males to have high perceptions compared to females while the previous study observed females to be more positive than males. The difference might be due to lecturers' behaviours towards students’ assessment activities. Aksu, Çivitçi and Duy (2008 cited in Yilmaz & Tinmaz [33], observed that students had no conviction in the fairness and objectivity offered by lecturers in measuring and evaluating their learning progress. Consequently, students had negative perceptions of the applications of measurement and assessment results.

### 4.5 Perceptions of students on lecturers’ teaching competencies based on sex and year of study when measured by indicators of technological competency

The study established the relationship between the variables as indicated in Table 9 below.

Table 8 shows that the p value for both sex and year of study (p > 0.05) is greater than 0.05. Therefore, the Null hypothesis is not be rejected. Since the P value is greater than 0.05 confidence level, there is no statistically significant relationship between the two means (P = 0.875, i.e., P > 0.05) for sex and (P = 0.949, i.e., P > 0.05) for the year of study. These findings show that there is no statistical relationship between male and female students' perceptions of lecturers' teaching competencies when measured by using technological competency as well as the number of years of study. Furthermore, the mean scores of male students was higher, i.e. (Mean = 77.45, standard deviation = 12.53, t = 0.612) compared to female students (Mean = 76.69, standard deviation = 12.08, t = 0.618). Again, the mean scores for 4th year students was slightly higher than the mean scores of 3rd year students, i.e., (4th year students' mean score = 77.29, Standard deviation = 11.86, t = −0.164 and 3rd year students' mean scores = 77.09, standard deviation = 12.69, t = −0.162). This indicates that male students do not share the same perceptions as female students; likewise, 4th year students do not share the same perceptions as 3rd year students. Each group has different perception of lecturers' teaching competencies as measured by technological competency. Female students exhibit lower perceptions compared to their male counterparts. This may be attributed to the limited number of female lecturers who can serve as role models, as well as family backgrounds that often encourage boys to pursue Science, Technology, Engineering, and Mathematics (STEM) courses while sidelining girls [[34], [35]]. Therefore, the perception of female students on technology remains to be low, whether they utilise Information, Communication and Technology (ICT) in their studies or lecturers integrate technology into the teaching process. This situation may persist unless deliberate efforts are made to address it. Consequently, female students tend to have lower perceptions of lecturers’ use of ICT compared to their male counterparts.

## 5 Conclusions and recommendations

### 5.1 Conclusions

This study concludes that students perceive the following dimensions as lecturers' teaching competencies that improve teaching and learning processes in public universities in Tanzania: lecturers' content competency, pedagogical competency, assessment and evaluation skills, and technological competency. Furthermore, the findings revealed no statistically significant relationship among the four dimensions based on sex and year of study. Each group of students had different perceptions. The differences in the findings occurred in sex and year of study are probably due to limited role of women who may be model to others in universities, limited number of lecturers, family background, lecturers' teaching behaviours, students’ beliefs and expectations and the maturity of the students (the longevity students stayed at university).

### 5.2 Recommendations

The findings of this study emphasize the importance of universities offering quality services, and one of the quality is competent lecturers. Under this digital world, modern lecturers should be aware of the needs of the customers and how to satisfy them. Through this, the universities are expected to produce products which will be capable of competing in a world market and be able to serve society. Thus, it is from this understanding that the study recommends universities employ more competent lecturers who can serve the purpose of teaching and learning processes. Furthermore, universities should continue offering professional development programmes to lecturers who are already employed to improve their technological and assessment skills. There should be a policy guideline prepared by the Tanzania Commission for Universities that whoever is employed as an academician in a university should undergo training on teaching competencies. This will help lecturers to have common understanding on the teaching competencies and to improve their teaching processes. Additionally, in order to maintain the quality of teaching and learning at universities, the directorate of quality assurance should monitor their activities through conducting a day to day classroom evaluation.

Moreover, further research should be conducted to determine how each indicator in four dimensions has significant relationship with students' perceptions on lecturers’ teaching competencies that improve teaching and learning processes in universities. In addition to that, similar study should be done in private universities to determine the perceptions of students on lecturers teaching competencies in Tanzania.

### 5.3 Limitation of the study

the study was limited to public universities in Tanzania. Therefore, findings of this study may not be generalised to the all universities found in Tanzania. Another limitation was anticipated due to the use of self-perceived questionnaires, which are known to have low response rates and potentially yield invalid data. These limitations were minimized by explaining the purpose of the study to the students and grouping them according to their program of study. Additionally, students were encouraged to seek clarification if they found any statements unclear, which was largely unnecessary due to the peer and expert reviews conducted prior to the data collection. With the support of their class representatives, we were able to efficiently collect all questionnaires with accurate data.

## Data availability statement

The data used in this study were part of previous research conducted during my PhD studies but were not included in the final thesis. (See https://repository.udom.ac.tz/bitstreams/a737ab80-eed9-45c0-a943-d9cdbc82c0f7/download). For access to the data, please contact the author directly.

## Ethics statement

The author Confirm that the study complies with all regulations.

I, the undersigned author of the above-mentioned study, hereby declare the following.

1. I have obtained written informed consent from the participant(s)/patient(s) for the publication of this study, any accompanying data and images. Where consent was obtained from someone other than the participant(s)/patient(s), I confirm that this proxy was authorised to provide consent on the participant's/patient's behalf.
2. Where the participant(s)/patient(s) is/are a minor(s), we followed local laws on the age and circumstances under which they may consent for themselves. If they were not of legal age to consent, consent was obtained from an authorised proxy i.e., the parents or legal guardian(s). If the minor(s) has/have reasonable understanding of the informed consent and implications, signature (or assent, as appropriate) was also obtained from the minor(s).
3. Where the participant(s)/patient(s) provided consent themselves, I confirm that they had capacity to do so, and any mental or physical disabilities were taken into consideration in the process of informing and obtaining written informed consent.
4. Where the participant(s)/patient(s) has/have died, I confirm that the consent given still allows for publication.
5. I confirm that all content presented in this study, associated data and images have been deidentified and anonymized to the best possible extent.
6. The original signed and dated consent form is held by the treating institution or appropriate governing local/regional/national body and will be retained according to the policies and procedures of the institution or governing body.
7. The written informed consent form (please **do not** include with your submission) includes all relevant information pertinent to each participant/patient (such as the name, age, condition, medical history, diagnosis, and treatment)
8. The participant(s)/patient(s)/authorized proxy were fully informed of the purpose of this study, the potential risks and benefits of publication, and the consequence of disclosing their personal information.
9. The participant(s)/patient(s) or authorized proxy were given the opportunity to ask questions regarding publication of the study, had their questions answered fully and have consented to publish all associated data and images. In the case of clinical studies, the participant(s)/patient(s) or authorized proxy approved the final version of the manuscript.
10. The participant(s)/patient(s) or legal guardian(s) were informed that their consent and participation in the publication of this study is entirely voluntary and that they have the right to withdraw their consent at any time.
11. If this is a clinical study manuscript, I confirm that at least one of the authors of this paper was involved in the care of the participant(s)/patient(s).
12. I confirm that my article complies with the appropriate local/regional/national law on consent and privacy.

By signing this declaration form, I acknowledge that I have read and understood the information provided above, and I attest to the accuracy of this declaration. I understand that any false or misleading information may result in the rejection of the manuscript or other disciplinary actions.

As corresponding author, I hereby declare that I sign this document on behalf of all the authors of the above-mentioned study involving human participants.

### Questionnaire for measuring lecturers’ teaching competencies

I am taking this opportunity to ask for your consent to participate in this study by filling in this questionnaire. Please note that the information you give will remain confidential and will be used for research purposes only. Efforts will be done to disseminate the findings of the current study through publications. Your cooperation in this discussion is highly anticipated.

Thank you for your acceptance to participate in this study.

#### Section a

Please put a tick in the space provided.

1. Sex. (i) Female … … … … … (ii) Male … … … … …
2. Studying Programme … … … … … … … … … … … … … … …
3. Year of study (i) … … … … … …(ii) … … … … … ….(iii) … … … … … …(iv) … … … ….

#### Section B

**Direction:** Please use the following scale to respond the following statements. Show your response by putting a tick to the box provided that describes your opinions on the extent to which your lecturer demonstrates that behaviour in the teaching and learning process.

Rating scale: 1 = Not at all, 2 = Very Little, 3 = A little, 4 = Much and 5 = Very Much.

**Table:** 

Statement	Not at all	Very little	A little	Much	Very much
My instructors possess up-to-date knowledge and skills in his/her subject matter.					
My instructors define prerequisite knowledge of the course from the outset.					
My instructors join students' previous learning experience with present knowledge.					
My instructors have ability to relate the course content to students' field of study.					
My instructors interpret the course contents from theoretical grounds to real life.					
My instructors transfer knowledge to real life.					
My instructors motivate students to be creative and innovative					

A: Indicators for Measuring Lecturers’ Content Competency.

B: Indicators for Measuring Lecturers’ Pedagogical Competency.

**Table:** 

Statements	Not at all	Very little	A Little	Much	Very Much
My instructors vary teaching methods to account for individual differences.					
My instructors determines teaching method suitable for one's course					
My instructors plans mode of presentation (lecture, seminar) according to students' characteristics					
My instructors chairs seminars and presentations					
My instructors are ready to learn from their students					
My instructors use modern learning methods to motivate continuous learning					
My instructors apply strategies of cooperative learning to encourage team work					
My instructors organizes the learning environment in the classroom in a way that reassures tranquility and induces feelings of security among students					

C: Indicators for Measuring Lecturers ‘Assessment and Evaluation Skills.

**Table:** 

Statements	Not at all	Very little	A Little	Much	Very Much
My instructors use varied evaluation methods suitable to behavioural objectives of the course					
My instructors are fair in grading of assignments and tests					
My instructors evaluate students’ through meaningful and authentic situations					
My instructors have capacity to provide timely feedback on assignments and tests					
My instructors assign suitable and enough time for evaluation					

D: Indicators for Measuring Lecturers’ Technological Competency.

**Table:** 

Statements	Not at all	Very little	A Little	Much	Very Much
My instructors use and employ ideas in scientific articles to improve one's course.					
My instructors Integrate information and communication technology in teaching and learning process.					
My instructors use technological and educational techniques to strengthen one's teaching skills and facilitate the process of learning					

## Funding statement

This research did not receive any specific grant from funding agencies in the public, commercial or not for profit sectors.

## Declaration of competing interest

The authors declare that they have no known competing financial interests or personal relationships that could have appeared to influence the work reported in this paper.
