# Supplementary material for: Lecturers' teaching competencies towards improving teaching and learning process in universities in Tanzania: Students’ perspectives
Source: Heliyon. 2025 Jan 3;11(1):e41683. doi: 10.1016/j.heliyon.2025.e41683 (PMC11760326; doi:10.1016/j.heliyon.2025.e41683)
Supplement: Multimedia component 1 [file mmc1.pdf]

Title of the study involving human participant(s): **Lecturers' Teaching Competencies towards Improving Teaching and Learning Process in Universities in Tanzania: Students' Perspectives**

Author Name(s): Asia Mbwebwe Rubeba

Institution(s): The University of Dodoma

Date: 26th, December, 2024

I, the undersigned author of the above-mentioned study, hereby declare the following:

1. I have obtained written informed consent from the participant(s) / patient(s) for the publication of this study, any accompanying data and images. Where consent was obtained from someone other than the participant(s) / patient(s), I confirm that this proxy was authorised to provide consent on the participant's / patient's behalf.
2. Where the participant(s) / patient(s) is/are a minor(s), we followed local laws on the age and circumstances under which they may consent for themselves. If they were not of legal age to consent, consent was obtained from an authorised proxy i.e., the parents or legal guardian(s). If the minor(s) has/have reasonable understanding of the informed consent and implications, signature (or assent, as appropriate) was also obtained from the minor(s).
3. Where the participant(s) / patient(s) provided consent themselves, I confirm that they had capacity to do so, and any mental or physical disabilities were taken into consideration in the process of informing and obtaining written informed consent.
4. Where the participant(s) / patient(s) has/have died, I confirm that the consent given still allows for publication.
5. I confirm that all content presented in this study, associated data and images have been deidentified and anonymized to the best possible extent.
6. The original signed and dated consent form is held by the treating institution or appropriate governing local / regional / national body and will be retained according to the policies and procedures of the institution or governing body.
7. The written informed consent form (please **do not** include with your submission) includes all relevant information pertinent to each participant / patient (such as the name, age, condition, medical history, diagnosis, and treatment)
8. The participant(s) / patient(s) / authorized proxy were fully informed of the purpose of this study, the potential risks and benefits of publication, and the consequence of disclosing their personal information.
9. The participant(s) / patient(s) or authorized proxy were given the opportunity to ask questions regarding publication of the study, had their questions answered fully and have consented to publish all associated data and images. In the case of clinical studies, the participant(s) / patient(s) or authorized proxy approved the final version of the manuscript.
10. The participant(s) / patient(s) or legal guardian(s) were informed that their consent and participation in the publication of this study is entirely voluntary and that they have the right to withdraw their consent at any time.
11. If this is a clinical study manuscript, I confirm that at least one of the authors of this paper was involved in the care of the participant(s) / patient(s).
12. I confirm that my article complies with the appropriate local / regional / national law on consent and privacy.

By signing this declaration form, I acknowledge that I have read and understood the information provided above, and I attest to the accuracy of this declaration. I understand that any false or misleading information may result in the rejection of the manuscript or other disciplinary actions.

As corresponding author, I hereby declare that I sign this document on behalf of all the authors of the above-mentioned study involving human participants.

Corresponding author's signature:	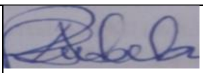
Date:	26th , December, 2024
